# Renoprotective effects of combination therapy with tolvaptan and dapagliflozin in autosomal dominant polycystic kidney disease: a four-case series

**DOI:** 10.1007/s13730-025-00990-7

**Published:** 2025-04-11

**Authors:** Junji Nishida, Mayuko Yamakawa, Shoko Miura, Masashi Yasutomi

**Affiliations:** Department of Nephrology, Kuwana City Medical Center, Kuwana, Japan

**Keywords:** Autosomal dominant polycystic kidney disease, Sodium-glucose cotransporter-2 inhibitor, Vasopressin V2-Receptor antagonist, Estimated glomerular filtration rate decline, Height-adjusted total kidney volume

## Abstract

Autosomal dominant polycystic kidney disease (ADPKD), the most prevalent inherited kidney disorder, progresses inexorably to end-stage kidney disease (ESKD) with the vasopressin V2-receptor antagonist tolvaptan serving as a primary treatment option since 2014. While dapagliflozin, a sodium-glucose cotransporter-2 (SGLT2) inhibitor, was approved for treating chronic kidney disease in August 2021, its renoprotective effects in ADPKD remain uncertain due to potential vasopressin stimulation. We evaluated four Japanese ADPKD patients receiving combination therapy with tolvaptan and dapagliflozin for over two years. A 74-year-old woman (Mayo Class 1D, CKD stage 4) showed improved estimated glomerular filtration rate (eGFR) decline from − 1.39 to − 0.66 mL/min/1.73 m^2^/year. A 62-year-old woman (Mayo Class 1B, CKD stage 3b) demonstrated eGFR decline improvement from − 1.02 to − 0.66 mL/min/1.73 m^2^/year. A 39-year-old man (Mayo Class 1C, CKD stage 3a) exhibited significant improvement from − 5.00 to − 1.35 mL/min/1.73 m^2^/year. A 45-year-old woman (Mayo Class 1D, CKD stage 3b) showed marked improvement from − 14.12 to − 0.22 mL/min/1.73 m^2^/year. While eGFR decline decelerated in the control group, the combination therapy group showed more pronounced improvements. Height-adjusted total kidney volume (htTKV) in the group combination therapy showed variable responses: two patients experienced volume increases (+ 4.03%, + 3.65%/year), while two showed decreases (− 0.45%, − 3.65%/year). These cases suggest potential renoprotective benefits from combining tolvaptan and dapagliflozin in ADPKD patients. Careful monitoring of renal cyst enlargement is warranted with concurrent dapagliflozin use. Further research is needed to confirm these preliminary findings and establish optimal patient selection criteria for combination therapy.

## Introduction

ADPKD represents the most common inherited kidney disorder worldwide, characterized by progressive development of bilateral renal cysts that can significantly impact multiple organ systems. In Japan, epidemiological data from 1994 revealed an ADPKD prevalence of approximately 1:4,000, with 50% of affected individuals progressing to end-stage kidney disease (ESKD) by age 60 [1]. Despite this significant disease burden, therapeutic options remain limited.

The vasopressin V2-receptor antagonist tolvaptan has served as the cornerstone of ADPKD management since its approval. However, ADPKD continues to rank among the leading causes of dialysis initiation as of 2022 [2], highlighting an urgent need for additional therapeutic approaches. The recent expansion of CKD treatment options through dapagliflozin's approval has generated interest in its potential application for ADPKD. Yet, major clinical trials investigating SGLT2 inhibitors in CKD, including DAPA-CKD [3] and EMPA-KIDNEY [4], specifically excluded ADPKD patients from their study populations. While Recommendation from the Japanese Society of Nephrology regarding the optimal application of SGLT2 inhibitors in CKD management [5], evidence supporting their efficacy in ADPKD remains scarce.

To address this knowledge gap, we present a detailed analysis of four ADPKD patients from our institution who received combination therapy with tolvaptan and dapagliflozin for more than two years. This case series examines their clinical characteristics, treatment responses, and outcomes, contributing to the emerging understanding of combination therapy in ADPKD management.

## Case presentation

**Fig. 1:**
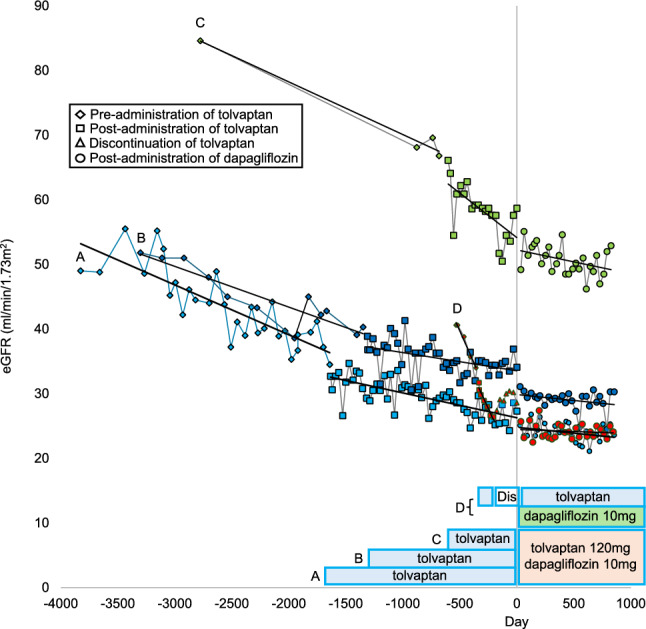
The progression in eGFR pre- and post- administration of tolvaptan, and post- administration of dapagliflozin (in the dapagliflozin group). *eGFR* estimated glomerular filtration rate, *Dis* discontinuation

**Fig. 2:**
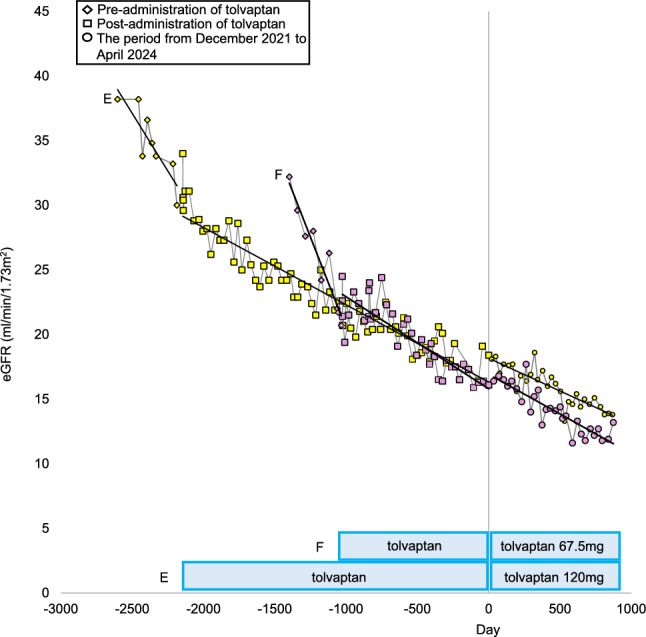
The progression in eGFR pre- and post- administration of tolvaptan and the period from December 2021 to April 2024 (in the control group). *eGFR* estimated glomerular filtration rate

**Fig. 3:**
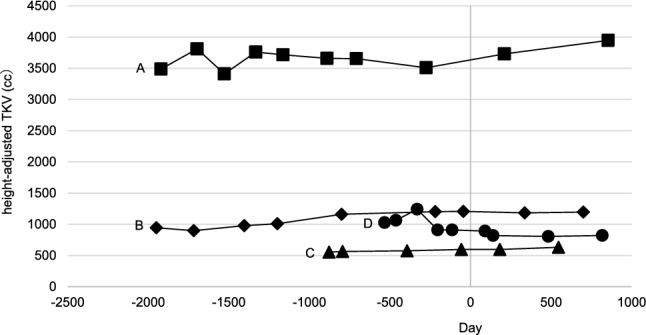
The progression of height adjusted TKV in the dapagliflozin group

**Fig. 4:**
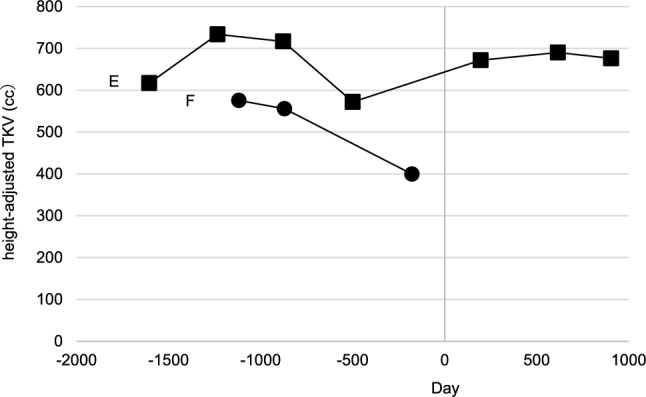
The progression of height adjusted TKV in the control group

We conducted a retrospective case series to evaluate the efficacy and safety of combination therapy with tolvaptan and dapagliflozin in patients with ADPKD. Between January 2017 and April 2024, we identified four patients (one male, three females: Case A, B, C, D) who received concurrent treatment with both medications at our institution. For comparison, we included two control patients with ADPKD (one male, one female: Case E, F) who received tolvaptan monotherapy for at least three years during the same period. All patients met the diagnostic criteria outlined in the Evidence-based clinical practice guideline for polycystic kidney disease (PKD) 2020 [6]. Baseline characteristics included demographic data and initial clinical measurements (Table 1). We analyzed eGFR trajectories (mL/min/1.73 m^2^) across three periods: pre-tolvaptan, post-tolvaptan, and post-dapagliflozin initiation (the period from December 2021 to April 2024 in the control group). The clinical course is presented in Figs. 1 and 2. The eGFR decline rate was determined using linear regression analysis, excluding the initial adaptation period (Table 2). The initial decline was specifically defined as the difference between baseline eGFR and the lowest eGFR value recorded within the first two months of dapagliflozin therapy, expressed both as an absolute value and percentage change from baseline. Additional monitoring included blood pressure (BP, mmHg), body mass index (BMI, kg/m^2^), Urinary protein-to-creatinine ratio (UPCR, mg/gCr), urinary osmolality (Uosm, mOsm/L), serum hemoglobin (Hb, g/dL), serum uric acid level (UA, mg/dL), and height-adjusted total kidney volume (htTKV, mL/m) (Table 3, 4, Fig. 3, 4). TKV measurements were derived from computed tomography scans using Ziostation2 volume measurement software (Ziosoft, Japan).

**Table 1:** Laboratory and clinical data at the initiation of dapagliflozin therapy

Group	Dapagliflozin				Control	
Case	A	B	C	D^※^	E	F
Sex	Female	Female	Male	Female	Female	Male
Age of dapagliflozin initiation / Age of reference date in the control (years)	74	62	39	45	50	74
Blood pressure	111/73	148/86	102/71	132/84	136/80	139/86
Body mass Index(kg/m2)	25.2	23.8	22.2	39	NA	29.1
Mayo Classification	1D	1B	1C	1D	1C	1B
Initiation date of tolvaptan	May 2017	Apr 2018	Apr 2020	Jan 2021	Jan 2016	Jan 2019
Initiation date of dapagliflozin / Reference date in the Control group	Nov 2021	Nov 2021	Dec 2021	Dec 2021	Nov 2021	Nov 2021
Duration of administration of dapagliflozin(day)	854	854	826	846	−	−
Laboratory Data at the Initiation of Dapagliflozin (Reference data in the Control group)						
Total kidney volume(ml)	5368	1840	1001	1373	938	651
Height-adjusted total kidney volume (ml/m)	3508	1202	595	909	572	399
Blood urea(mg/dl)	19.1	17.8	14.8	18.1	33.9	43.7
Creatine(mg/dl)	1.47	1.26	1.14	1.53	2.34	3.14
eGFR(ml/min/1.73m2)	27.3	34.1	58.7	30.2	18.4	16.1
Initial decline in eGFR (%)	8.0	8.8	16.2	9.6	−	−
Hemoglobin(g/dl)	10.9	10.3	13	12.4	10.4	11.2
uric acid(mg/dl)	6.8	5.8	6.7	4.1	3.8	4
HbA1C (%)	6	5.5	5.9	5.9	5.6	5.7
Plasma glucose(mg/dl)	98	140	102	104	101	154
Urinary protein(mg/dl)	424	65	NA	NA	35	264
Urine osmolality(mOsm/L)	173	90	121	351	134	231
Complications						
Liver cysts	+	+	+	+	+	+
Hypertension	+	+	+	+	+	+
Intracranial aneurysm	−	−	−	−	−	+
Diabetes	−	−	−	−	−	−
Medications						
Tolvaptan, dosage(mg)	120	120	120	0	120	67.5
RAS inhibitor	+	+	+	+	+	+

*eGFR* estimated glomerular filtration rate, *HbA1C* hemoglobin A, *NA* not available, *Uosm* Urine osmolality, *ARB* angiotensin receptor blocker

※In Patient D, tolvaptan therapy was resumed one month after the initiation of dapagliflozin. Type 2 diabetes mellitus was diagnosed 19 months following the start of dapagliflozin treatment, after which semaglutide therapy was introduced

**Table 2:** Annual change in estimated glomerular filtration decline rate (eGRF decline rate) for each period in the dapagliflozin (Case A-D) and the control groups (Case E, F) and change in the eGFR decline rate during the post-tolvaptan period and the post-dapagliflozin period

Case	Pre-tolvaptan (ml/min/1.73m^2^/year)	Post-tolvaptan (ml/min/1.73m^2^/year)	Post-dapagliflozin (ml/min/1.73m^2^/year)	ΔeGFR decline rate (ml/min/1.73m^2^/year, %)	The percentage change in eGFR decline rate (%)
A	-2.81	-1.39	-0.66	0.73	52.5
B	-2.30	-1.02	-0.66	0.36	35.0
C	-2.96	-5.00	-1.35	3.65	73.0
D	-14.53	-14.12	-0.22	13.90	98.4
Case	Pre-tolvaptan (ml/min/1.73m^2^/year)	Post-tolvaptan (ml/min/1.73m^2^/year)	Since Dec 2021 (ml/min/1.73m^2^/year)	ΔeGFR decline rate (ml/min/1.73m^2^/year, %)	The percentage change in eGFR decline rate (%)
E	-6.53	-2.19	-1.86	0.33	15.1
F	-10.29	-2.48	-2.23	0.25	10.1

ΔeGFR decline rate = Post-dapagliflozin − Post-tolvaptan; The percentages change in eGFR decline rate was calculated as (Post-dapagliflozin − Post-tolvaptan) / Post-tolvaptan × 100%

**Table 3:** Change in mean hemoglobin concentration, uric acid, and urine osmolality one year pre- and post- dapagliflozin administration

	Case	Pre-dapagliflozin	Post-dapagliflozin	P-value
Hemoglobin (g/dl)	A	11.2 ± 0.2	11.7 ± 0.3	< 0.001
	B	10.8 ± 0.3	12.0 ± 0.5	< 0.001
	C	12.8 ± 0.4	13.1 ± 0.4	0.084
	D	12.0 ± 0.4	12.3 ± 0.5	< 0.05
	Case	Up to November 2021	Since December 2021	P-value
	E	9.9 ± 0.7	10.7 ± 0.4	< 0.05
	F	10.8 ± 0.5	10.1 ± 1.6	0.155
	Case	Pre-dapagliflozin	Post-dapagliflozin	P-value
Uric acid (mg/dl)	A	6.7 ± 0.7	5.6 ± 0.5	< 0.001
	B	5.9 ± 0.5	5.7 ± 0.5	0.263
	C	7.0 ± 0.6	5.1 ± 0.3	< 0.001
	D	6.5 ± 1.0	5.8 ± 0.9	0.052
	Case	Up to November 2021	Since December 2021	P-value
	E	3.7 ± 0.2	4.3 ± 0.5	0.235
	F	5.2 ± 0.6	3.9 ± 0.5	< 0.001
	Case	Pre-dapagliflozin	Post-dapagliflozin	P-value
Urine osmolality (mOsm/kg H2O)	A	110.8 ± 13.4	126.1 ± 17.5	< 0.001
	B	168.1 ± 42.4	163.4 ± 43.0	0.642
	C	136.5 ± 23.3	161.5 ± 29.0	< 0.001
	D	88.3 ± 20.0	136.8 ± 25.7	< 0.001
	Case	Up to November 2021	Since December 2021	P-value
	E	128.9 ± 9.5	136.8 ± 10.8	0.093
	F	195.3 ± 52.8	249.5 ± 58.2	< 0.05

**Table 4:** Annual Changes in Blood Pressure, Body Mass Index, and Urinary Protein to Creatine Ratio

	Case	Pre-dapagliflozin (1 Year Before Baseline)	Pre-dapagliflozin (Baseline)	Post-dapagliflozin (1 Year After Baseline)	Post-dapagliflozin (2 Years After Baseline)
Blood Pressure	A	127/78	111/73	134/83	135/67
(mmHg)	B	138/84	148/86	140/85	137/87
	C	130/72	102/71	117/60	103/63
	D	141/93	132/84	128/80	121/78
	E	133/54	126/55	135/62	137/81
	F	139/85	139/86	150/84	137/76
Body Mass Index	A	25.9	25.2	24.6	23.7
(kg/m^2^)	B	23.5	23.8	23.4	22.9
	C	24.0	22.2	21.2	21.4
	D	38.4	40.1	40.3	34.4
	E	17.1	NA	15.6	NA
	F	29.8	29.1	28.6	NA
Urinary Protein to	A	270	424	69	47
Creatine Ratio	B	71	65	83	81
(mg/gCr)	C	NA	NA	56	92
	D	104	NA	NA	NA
	E	41	35	26	40
	F	61	264	369	164

*NA* not available

For statistical analysis, we compared mean laboratory values before and after dapagliflozin administration using paired t-tests. The pre-dapagliflozin period was defined as the year preceding treatment initiation, while the post-dapagliflozin period encompassed the first year of treatment. All laboratory values are presented as mean ± standard deviation, with statistical significance set at p < 0.05. Statistical analyses were performed using EZR, a modified version of R commander [7].

Case A: A 74-year-old Japanese woman with CKD stage 4 and sporadic Mayo Class 1D ADPKD showed an improvement in her eGFR decline rate from − 2.81 to − 1.39 mL/min/1.73m^2^/year with tolvaptan, further improving to − 0.66 mL/min/1.73m^2^/year after 854 days of combination therapy. HtTKV increased from 3508 to 3946 cc, representing an annual growth rate of 4.03%. One-year follow-up revealed an increase in Uosm, along with improvements in both Hb and UA. Her BP increased, while BMI and UPCR decreased.

Case B: A 62-year-old Japanese woman with CKD stage 3b and Mayo Class 1B ADPKD, with a documented family history of the condition, showed an improvement in her eGFR decline rate from − 2.30 to − 1.02 mL/min/1.73m^2^/year with tolvaptan, further improving to − 0.66 mL/min/1.73 m^2^/year after 854 days of combination therapy. HtTKV decreased from 1207 to 1196 cc, representing a reduction of 0.45%/year. One-year follow-up revealed a slight decrease in Uosm, improvements in Hb, and a slight decrease in UA. Her BP and BMI showed a downward trend.

Case C: A 39-year-old man, the son of Case B, with CKD stage 3a and Mayo Class 1C ADPKD showed eGFR decline from − 2.96 to − 5.00 mL/min/1.73m^2^/year with tolvaptan. Over the subsequent 826 days of combination therapy, his eGFR decline rate improved to -1.35 mL/min/1.73 m^2^/year. HtTKV increased from 596 to 632 cc, representing an annual growth rate of 3.65%. One-year follow-up revealed increase in Uosm, slight improvements in Hb, a notable decrease in UA.

Case D: A 45-year-old Japanese woman presented with CKD stage3b and sporadic Mayo Class 1D ADPKD. Five months after initiating tolvaptan, she developed liver dysfunction, leading to the discontinuation of tolvaptan. After her liver dysfunction improved, tolvaptan was resumed one month after dapagliflozin administration, with dose escalations to 90 mg at five months and 120 mg at 25 months. At 19 months after dapagliflozin initiation, the patient developed type 2 diabetes and began semaglutide treatment, which resulted in a 10-kg reduction in body weight. Her eGFR decline rate almost unchanged with tolvaptan. After 846 days of dapagliflozin treatment, her eGFR decline rate improved to − 0.22 mL/min/1.73 m^2^/year. HtTKV decreased from 905 to 821 cc, representing a reduction of 3.65%/year. One-year follow-up revealed an increase in Uosm, improvement in Hb, a slight decrease in UA. Her BP showed a downward trend,

Case E: A 50-year-old woman with CKD stage 3b and familial Mayo Class 1C ADPKD, who received tolvaptan monotherapy, showed eGFR decline improvement from − 6.53 to − 2.19 mL/min/1.73m^2^/year. After 847 days from December 2021, the eGFR decline rate was − 1.86 mL/min/1.73 m^2^/year. HtTK remained stable (Fig. 4). One-year follow-up revealed a slight increase in Uosm, improvements in Hb, a slight increase in UA.

Case F: A 74-year-old man with CKD stage 4 and familial Mayo Class 1C ADPKD, who received tolvaptan monotherapy showed eGFR decline improvement from − 10.29 to − 2.48 mL/min/1.73m^2^/year. After 839 days from December 2021, the eGFR decline rate was − 2.23 mL/min/1.73 m^2^/year. HtTK was not assessed. One-year follow-up revealed an increase in Uosm, a slight decrease in Hb, and decrease in UA.

Adverse events after initiation of dapagliflozin included the development of pyelonephritis in Cases A and B, both of whom achieved complete resolution following antibiotic therapy.

## Discussion

The addition of dapagliflozin to tolvaptan therapy demonstrates promising renoprotective potential in ADPKD patients, evidenced by reduced eGFR decline rates, although the observed increases in kidney volume in some patients necessitate careful monitoring. SGLT2 specifically handles 90% of glucose reabsorption in renal proximal tubules [8, 9]. SGLT2 inhibitors exert renoprotective effects in both diabetic and non-diabetic CKD patients by reducing intraglomerular pressure, optimizing glycemic control, decreasing body weight, ameliorating anemia, and reducing plasma uric acid levels [3, 4, 10, 13–15]. These agents decrease metabolic overload through reduced Na + -glucose reabsorption and improved mitochondrial function, while simultaneously inducing osmotic diuresis and providing anti-inflammatory benefits [10–12].

Despite these established mechanistic benefits, studies of SGLT2 inhibitors in ADPKD animal models have yielded inconsistent results [8, 16, 17]. A key consideration is the potential increase in vasopressin levels following SGLT2 inhibitor administration, which could theoretically accelerate ADPKD progression [18].

Clinical evidence regarding SGLT2 inhibition in ADPKD remains mixed. In a case series involving 20 ADPKD patients treated with dapagliflozin, Morioka et al. [19] observed a decline in eGFR along with an increase in TKV following dapagliflozin therapy. Similarly, Nakatani et al. [20] reported deterioration in eGFR and TKV in patients administered dapagliflozin without concurrent tolvaptan treatment.

Our findings of slower eGFR decline align with previous studies by Yoshimoto et al. [22] and Minatoguchi et al. [21], who reported renoprotective effects of dapagliflozin in tolvaptan-treated ADPKD patients. In Cases C and D, the addition of tolvaptan did not improve the eGFR decline rate; however, a notable improvement was observed following the introduction of dapagliflozin. Moreover, our findings revealed that while the eGFR decline slowed in the control group, the reduction in decline was notably more pronounced in the group receiving dapagliflozin (Table 2). The observation of benefits across Mayo Classification classes 1B-1D suggests that renoprotective effects occur independently of disease severity.

The increased urinary osmolality observed in three of four patients suggests elevated vasopressin levels following dapagliflozin administration. However, this elevation may not indicate poor outcomes in tolvaptan-treated patients. Gansevoort et al. [23] demonstrated that higher copeptin levels in tolvaptan-treated patients correlated with better disease outcomes, including reduced renal enlargement and higher eGFR at three years.

The observed htTKV increases in two patient’s parallel findings from Yoshimoto et al. [22]. This increase likely results from compensatory glucose reabsorption in the proximal tubular S3 segment [24, 25], potentially promoting cyst enlargement through mTOR activation and AMPK inactivation [25, 26]. SGLT2 inhibition may also upregulate HIF-1α in collecting ducts [25], possibly contributing to cyst expansion [27].

Our study's primary limitations include its small sample size and retrospective design, which preclude definitive conclusions. Treatment varied among patients, particularly in Case D, who temporarily discontinued tolvaptan and later initiated semaglutide. The absence of vasopressin measurements and genetic confirmation between Cases B and C constrains our understanding of the underlying mechanisms.

Further investigation should focus on larger, prospective studies with control groups to characterize the effects of combined therapy in ADPKD. Examination of long-term outcomes and potential synergistic effects with other renoprotective agents, particularly GLP-1 receptor agonists like semaglutide [28], could optimize ADPKD management strategies.

In conclusion, while the addition of dapagliflozin to tolvaptan therapy demonstrates promising renoprotective effects through slower eGFR decline, the potential for increased kidney volume necessitates careful monitoring. These findings contribute to the growing evidence supporting SGLT2 inhibitor use in ADPKD management, though larger prospective studies are essential to evaluate long-term outcomes of combination therapy.

- Disclosure of potential conflicts of interest: The authors have declared no competing interests.
- Research involving Human Participants and/or Animals: This article does not contain any studies with human participants performed by any of the authors.
- Informed consent: Written informed consent was obtained from all the patients described in the present case.
